# *meso*-Dimethyl 2,5-dibromo­hexa­ne­dioate

**DOI:** 10.1107/S1600536810045642

**Published:** 2010-11-13

**Authors:** Zhi-Qiang Feng, Yuan-Feng Ye, Xiao-Li Yang, Tao Dong, Huai-Qing Wang

**Affiliations:** a School of Material Engineering, Jinling Institute of Technology, Nanjing 211169, People’s Republic of China

## Abstract

The title compound, C_8_H_12_Br_2_O_4_, lies about a crystallographic center of inversion at the midpoint of the central C—C bond. The latter is also repsonsible for the observation of the *meso* form. There are no intra­molecular hydrogen bonds, but mol­ecules are connected by inter­molecular C—H⋯O inter­actions, forming a three-dimensional network.

## Related literature

The title compound is an important intermediate in organic synthesis. For the synthetic procedure, see: McDonald & Reitz (1972 ▶). For bond-length data, see: Allen *et al.* (1987 ▶).
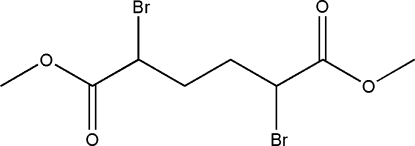


## Experimental

### 

#### Crystal data

C_8_H_12_Br_2_O_4_
*M* *_r_* = 331.98
Monoclinic, 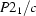

*a* = 4.5580 (9) Å
*b* = 12.134 (2) Å
*c* = 10.554 (2) Å
β = 90.36 (3)°
*V* = 583.7 (2) Å^3^
*Z* = 2
Mo *K*α radiation
μ = 6.93 mm^−1^
*T* = 293 K
0.20 × 0.10 × 0.10 mm

#### Data collection

Enraf–Nonius CAD-4 diffractometer
Absorption correction: ψ scan (North *et al.*, 1968 ▶) *T*_min_ = 0.338, *T*_max_ = 0.544
1428 measured reflections
1271 independent reflections
639 reflections with *I* > 2σ(*I*)
*R*_int_ = 0.071
3 standard reflections every 200 reflections  intensity decay: 1%

#### Refinement

*R*[*F* ^2^ > 2σ(*F* ^2^)] = 0.040
*wR*(*F* ^2^) = 0.072
*S* = 1.00
1271 reflections
64 parameters
3 restraints
H-atom parameters constrained
Δρ_max_ = 0.33 e Å^−3^
Δρ_min_ = −0.37 e Å^−3^

### 

Data collection: *CAD-4 Software* (Enraf–Nonius, 1985 ▶); cell refinement: *CAD-4 Software*; data reduction: *XCAD4* (Harms & Wocadlo, 1995 ▶); program(s) used to solve structure: *SHELXS97* (Sheldrick, 2008 ▶); program(s) used to refine structure: *SHELXL97* (Sheldrick, 2008 ▶); molecular graphics: *SHELXTL* (Sheldrick, 2008 ▶); software used to prepare material for publication: *SHELXTL*.

## Supplementary Material

Crystal structure: contains datablocks I, global. DOI: 10.1107/S1600536810045642/im2243sup1.cif

Structure factors: contains datablocks I. DOI: 10.1107/S1600536810045642/im2243Isup2.hkl

Additional supplementary materials:  crystallographic information; 3D view; checkCIF report

## Figures and Tables

**Table 1:** Hydrogen-bond geometry (Å, °)

*D*—H⋯*A*	*D*—H	H⋯*A*	*D*⋯*A*	*D*—H⋯*A*
C3—H3*A*⋯O2^i^	0.98	2.59	3.33 (1)	132

Symmetry code: (i) 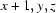.

**Fig. 1.**
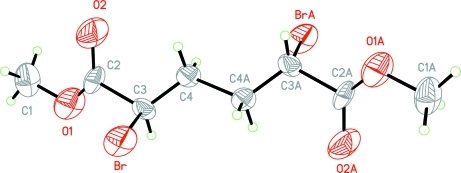
Molecular structure of the title compound showing the atom-numbering scheme. Displacement ellipsoids are drawn at the 40% probability level. Atoms labeled with the suffixes A are generated by the symmetry operation (1/2 - x, 3/2 - y, 1 - z). Hydrogen bonds are shown as dashed lines.

**Fig. 2.**
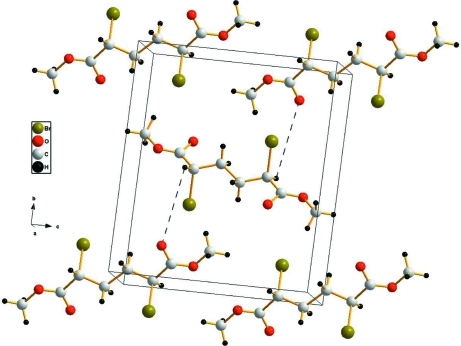
Packing diagram for (I). C—H···O hydrogen bonds are shown as dashed lines.

## supplementary crystallographic information

### Comment

The tittle compound, *meso*-2,5-dibromo-hexanedioic acid dimethyl ester is an important intermediate for the synthesis of dimethyl cyclobut-1-ene-1,2-dicarboxylate. We herein report the crystal structure of the title compound, (I).

The molecular structure of (I) is shown in Fig. 1. Bond lengths and angles are within normal ranges (Allen *et al.*, 1987).

The central C4—C4A bond of the title compound, C_8_H_12_Br_2_O_4_, represents a crystallographic center of inversion. The latter is also repsonsible for the observation of the *meso* form. There are no intramolecular hydrogen bonds, but molecules of the title compound are connected by C—H···O intermolecular interactions to form a three dimensional network (Table 1).

### Experimental

The title compound, (I) was prepared by a method reported in literature (McDonald & Reitz, 1972). Single crystals were obtained by dissolving (I) (0.5 g, 1.5 mmol) in ethanol (25 ml) and evaporating the solvent slowly at room temperature for about 3 d.

### Refinement

H atoms were positioned geometrically, with C—H = 0.96 Å for alkyl H, and constrained to ride on their parent atoms, with *U*_iso_(H) = 1.5*U*_eq_(C).

### Crystal data

**Table:**

C_8_H_12_Br_2_O_4_	*F*(000) = 324
*M**_r_* = 331.98	*D*_x_ = 1.889 Mg m^−^^3^
Monoclinic, *P*2_1_/*c*	Mo *K*α radiation, λ = 0.71073 Å
Hall symbol: -P 2ybc	Cell parameters from 25 reflections
*a* = 4.5580 (9) Å	θ = 9–13°
*b* = 12.134 (2) Å	µ = 6.93 mm^−^^1^
*c* = 10.554 (2) Å	*T* = 293 K
β = 90.36 (3)°	Block, colourless
*V* = 583.7 (2) Å^3^	0.20 × 0.10 × 0.10 mm
*Z* = 2	

### Data collection

**Table:**

Enraf–Nonius CAD-4 diffractometer	639 reflections with *I* > 2σ(*I*)
Radiation source: fine-focus sealed tube	*R*_int_ = 0.071
graphite	θ_max_ = 27.1°, θ_min_ = 2.6°
ω/2θ scans	*h* = 0→5
Absorption correction: ψ scan (North *et al.*, 1968)	*k* = −15→0
*T*_min_ = 0.338, *T*_max_ = 0.544	*l* = −13→13
1428 measured reflections	3 standard reflections every 200 reflections
1271 independent reflections	intensity decay: 1%

### Refinement

**Table:**

Refinement on *F*^2^	Primary atom site location: structure-invariant direct methods
Least-squares matrix: full	Secondary atom site location: difference Fourier map
*R*[*F*^2^ > 2σ(*F*^2^)] = 0.040	Hydrogen site location: inferred from neighbouring sites
*wR*(*F*^2^) = 0.072	H-atom parameters constrained
*S* = 1.00	*w* = 1/[σ^2^(*F*_o_^2^) + (0.022*P*)^2^] where *P* = (*F*_o_^2^ + 2*F*_c_^2^)/3
1271 reflections	(Δ/σ)_max_ < 0.001
64 parameters	Δρ_max_ = 0.33 e Å^−^^3^
3 restraints	Δρ_min_ = −0.37 e Å^−^^3^

### Special details

**Table:**

Geometry. All e.s.d.'s (except the e.s.d. in the dihedral angle between two l.s. planes) are estimated using the full covariance matrix. The cell e.s.d.'s are taken into account individually in the estimation of e.s.d.'s in distances, angles and torsion angles; correlations between e.s.d.'s in cell parameters are only used when they are defined by crystal symmetry. An approximate (isotropic) treatment of cell e.s.d.'s is used for estimating e.s.d.'s involving l.s. planes.
Refinement. Refinement of *F*^2^ against ALL reflections. The weighted *R*-factor *wR* and goodness of fit *S* are based on *F*^2^, conventional *R*-factors *R* are based on *F*, with *F* set to zero for negative *F*^2^. The threshold expression of *F*^2^ > σ(*F*^2^) is used only for calculating *R*-factors(gt) *etc*. and is not relevant to the choice of reflections for refinement. *R*-factors based on *F*^2^ are statistically about twice as large as those based on *F*, and *R*- factors based on ALL data will be even larger.

### Fractional atomic coordinates and isotropic or equivalent isotropic displacement parameters (Å^2^)

**Table:**

	*x*	*y*	*z*	*U*_iso_*/*U*_eq_	
Br	0.23822 (12)	0.64074 (4)	0.70596 (6)	0.0813 (2)	
O1	0.3762 (8)	0.4221 (3)	0.8857 (4)	0.0888 (12)	
O2	0.0821 (9)	0.3620 (3)	0.7400 (4)	0.0980 (12)	
C1	0.2255 (11)	0.3583 (4)	0.9715 (5)	0.0872 (17)	
H1A	0.3263	0.3595	1.0516	0.131*	
H1B	0.2144	0.2839	0.9409	0.131*	
H1C	0.0310	0.3872	0.9817	0.131*	
C2	0.2723 (12)	0.4259 (4)	0.7836 (5)	0.0572 (12)	
C3	0.4550 (10)	0.4973 (3)	0.6885 (4)	0.0477 (11)	
H3A	0.6595	0.5044	0.7166	0.057*	
C4	0.4369 (9)	0.4614 (3)	0.5570 (4)	0.0475 (11)	
H4A	0.2315	0.4476	0.5384	0.057*	
H4B	0.5370	0.3911	0.5517	0.057*	

### Atomic displacement parameters (Å^2^)

**Table:**

	*U*^11^	*U*^22^	*U*^33^	*U*^12^	*U*^13^	*U*^23^
Br	0.0997 (4)	0.0232 (2)	0.1214 (5)	0.0072 (3)	0.0337 (3)	−0.0032 (4)
O1	0.111 (3)	0.058 (2)	0.098 (3)	0.002 (2)	0.007 (2)	0.035 (2)
O2	0.119 (3)	0.063 (3)	0.112 (3)	−0.028 (3)	0.023 (2)	0.020 (3)
C1	0.123 (5)	0.061 (4)	0.078 (3)	0.014 (4)	0.029 (3)	0.022 (3)
C2	0.062 (3)	0.040 (3)	0.070 (3)	0.006 (3)	0.022 (2)	0.025 (3)
C3	0.070 (3)	0.029 (2)	0.043 (2)	−0.002 (2)	−0.0005 (19)	−0.0068 (19)
C4	0.063 (3)	0.029 (2)	0.050 (3)	−0.004 (2)	−0.006 (2)	0.007 (2)

### Geometric parameters (Å, °)

**Table:**

Br—C3	2.011 (4)	C2—C3	1.569 (6)
O1—C2	1.175 (5)	C3—C4	1.456 (5)
O1—C1	1.378 (5)	C3—H3A	0.9800
O2—C2	1.249 (6)	C4—C4^i^	1.632 (7)
C1—H1A	0.9600	C4—H4A	0.9700
C1—H1B	0.9600	C4—H4B	0.9700
C1—H1C	0.9600		
			
C2—O1—C1	115.1 (5)	C4—C3—Br	108.7 (3)
O1—C1—H1A	109.5	C2—C3—Br	99.0 (3)
O1—C1—H1B	109.5	C4—C3—H3A	111.3
H1A—C1—H1B	109.5	C2—C3—H3A	111.3
O1—C1—H1C	109.5	Br—C3—H3A	111.3
H1A—C1—H1C	109.5	C3—C4—C4^i^	120.9 (4)
H1B—C1—H1C	109.5	C3—C4—H4A	107.1
O1—C2—O2	126.0 (5)	C4^i^—C4—H4A	107.1
O1—C2—C3	113.3 (5)	C3—C4—H4B	107.1
O2—C2—C3	118.5 (5)	C4^i^—C4—H4B	107.1
C4—C3—C2	114.7 (4)	H4A—C4—H4B	106.8
			
C1—O1—C2—O2	−15.8 (8)	O1—C2—C3—Br	−95.3 (4)
C1—O1—C2—C3	−178.7 (4)	O2—C2—C3—Br	100.4 (4)
O1—C2—C3—C4	149.2 (4)	C2—C3—C4—C4^i^	168.8 (4)
O2—C2—C3—C4	−15.1 (6)	Br—C3—C4—C4^i^	59.1 (5)

Symmetry codes: (i) −*x*+1, −*y*+1, −*z*+1.

### Hydrogen-bond geometry (Å, °)

**Table:**

*D*—H···*A*	*D*—H	H···*A*	*D*···*A*	*D*—H···*A*
C3—H3A···O2^ii^	0.98	2.59	3.33 (1)	132

Symmetry codes: (ii) *x*+1, *y*, *z*.
